# Alteration of Bumblebee Venom Composition toward Higher Elevation

**DOI:** 10.3390/toxins12010004

**Published:** 2019-12-19

**Authors:** Nezahat Pınar Barkan, Mathieu Chevalier, Jean-Nicolas Pradervand, Antoine Guisan

**Affiliations:** 1 Department of Ecology and Evolution, University of Lausanne, CH-1015 Biophore, Lausanne, Switzerland; pinarbarkan@gmail.com (N.P.B.); mathieu.chevalier@unil.ch (M.C.); 2 Swiss Ornithological Institute, Valais Field Station, Rue du Rhône 11, CH-1950 Sion, Switzerland; jean-nicolas.pradervand@vogelwarte.ch; 3 Institute of Earth Surface Dynamics, University of Lausanne, CH-1015 Géopolis, Lausanne, Switzerland

**Keywords:** venom, bumblebee, elevation, shot-gun proteomics, mixed-effect model, PLA_2_-like

## Abstract

Venomous animals use venom, a complex biofluid composed of unique mixtures of proteins and peptides, for either predation or defense. Bumblebees, which occur in various habitats due to their unique thermoregulatory properties, mainly use venom for defense. Herein, we conducted an exploratory analysis of the venom composition of a bumblebee species (*Bombus pascuorum*) along an elevation gradient in the western Swiss Alps using shot-gun proteomic approaches to assess whether their defense mechanism varies along the gradient. The gradient was characterized by high temperatures and low humidity at low elevations and low temperatures and high humidity at high elevations. Venom composition is changing along the elevation gradient, with proteomic variation in the abundances of pain-inducing and allergenic proteins. In particular, the abundance of phospholipase A_2_-like, the main component of bumblebee venom, gradually decreases toward higher elevation (lower temperature), suggesting venom alteration and thus a decrease in bumblebee defense towards harsher environments. Larger datasets may complement this study to validate the observed novel trends.

## 1. Introduction

Venom is a complex bio-fluid secreted by the host venom gland. It is composed of unique mixtures of proteins and peptides that act on the vital system of preys or predators [1]. Venomous animals are distributed in all phyla of the animal kingdom, encompassing more than 100,000 species displaying multiple functions [2]. While the most characterized function of venom is predation, it can also be used for competition [3,4], reproduction [5], and defense [4,5]. Venom is an example of convergent evolution and has been shown to have evolved independently in different taxa owing to the strength of particular selective pressures [6,7,8]. For instance, in predation, venom is expected to be positively selected when resources are limited because it confers a competitive advantage to predators [9]. On the other hand, in defense, venom is positively selected when predators are abundant because it confers a competitive advantage to preys [10,11]. These selective pressures have fostered changes in venom composition [4], resulting in adaptations to different environments, in turn leading to niche partitioning [12,13]. As a result, venom is now frequently considered as a phenotypic response to changes in predator/prey interactions or habitat features [14,15]. Accordingly, several studies conducted on a range of predatory animals have shown that venom composition can strongly vary along ecological gradients including dietary pressures [16,17,18], but also climatic variables [15,19,20,21,22]. The extent to which this plastic response holds for defensive animals has, however, remained largely unexplored.

Bumblebees belong to the order Hymenoptera, a diverse group with members exhibiting different life-history strategies and inhabiting different ecological niches [23,24]. They have a worldwide distribution and are commonly found in alpine, subalpine, and arctic zones [25]. Bumblebees, which are the closest relatives of honeybees (*Apis* sp.), are unique among bees because they are able to reach high altitudes due to their unique thermoregulatory properties which enable them to tolerate various climatic conditions [26,27,28]. There are about 250 species of bumblebees estimated in the world [29], 30 of which can be found in the western Swiss Alps [27]. Bumblebees play an essential role in the functioning of alpine and agricultural ecosystems, acting as important pollinators of wild and cultivated plants [30]. In recent years, a widespread decline of bumblebees has, however, been observed globally [31]. Furthermore, they are prone to be threatened by climate-mediated changes predicted to occur in mountainous regions [32,33].

Bumblebees solely use venom for defensive purposes, similarly to other animals like honeybees, sea urchins, and venomous fishes [34]. Compared with predatory venoms, defensive venoms are simpler in composition and have a direct and immediate effect on physiological processes [4,35]. Defensive venoms are mostly directed against predators and have been shown to cause paralysis and instant pain [4], owing to the presence of different allergenic elements within the venom profile. In bumblebees, these components include enzymes (phospholipase A_2_, hyaluronidase), peptides (mast cell degranulating peptide and bombolitin), acid phosphatases, proteases, and protease inhibitors [36,37,38]. Given the numerous food resources that are associated to bumblebees (e.g., honey, pollen, larvae, or pupae [39]), these organisms usually face strong predation pressures (e.g., mammals, reptiles, amphibians, birds, or moths), stressing the importance of these defensive mechanisms for their survival [40].

Although few studies have been conducted regarding the venom plasticity of defensive animals, several biotic and abiotic factors have been shown to have an influence on the venom composition of predatory venomous animals. For these predatory animals, while diet seems to be the major driver of venom evolution as it is a foraging adaptation [41], some studies have shown that venom composition can also vary depending on some environmental factors. For instance, snake venom composition has been shown to vary depending on site elevation and potentially, temperature [15,20,22]. Similarly, temperature has been shown to impact the venom sac transcriptome of the sea anemone [19]. Altogether, these studies highlight the dynamic nature of venom composition in animals with frequent encounters with their preys, the selective pressures that act upon venom composition [4]. The impact of environmental factors on defensive venom composition has, to our knowledge, been limited to only one study, showing a synchronized seasonal variation in the levels of PLA_2_ and melittin, a honeybee-specific toxin [42]. However, other types of changes may be considered. For instance, climatic conditions are expected to have an impact on predator–prey interactions, and some studies have shown that the frequency and the strength of biotic interactions tend to be more important at low elevations characterized by high temperatures [43,44,45]. Based on this and given the metabolic cost of venom production [46], one could expect a decrease in the abundance of defense-related toxins along the elevation gradient due to lower predation pressures. In support for this hypothesis, scorpion venom composition has been shown to vary in response to simulated predator exposure, with a higher production of predator-active defensive toxins and a decrease in predatory compounds directed against their prey [14].

Here, we ran an exploratory study to investigate the extent to which the composition of bumblebee venom varies along an elevation gradient in the western Swiss Alps. We hypothesized that venom composition in bumblebees might vary considerably along the elevation gradient owing to changes in environmental conditions and/or through decreased predation pressure [40]. We assessed this by exploring the variation of putative venom toxins in one widespread species, *Bombus pascuorum*, along an elevation gradient.

## 2. Results

### 2.1. The Venom Composition of B. pascuorum Varies along the Elevation Gradient

**Figure 1:**
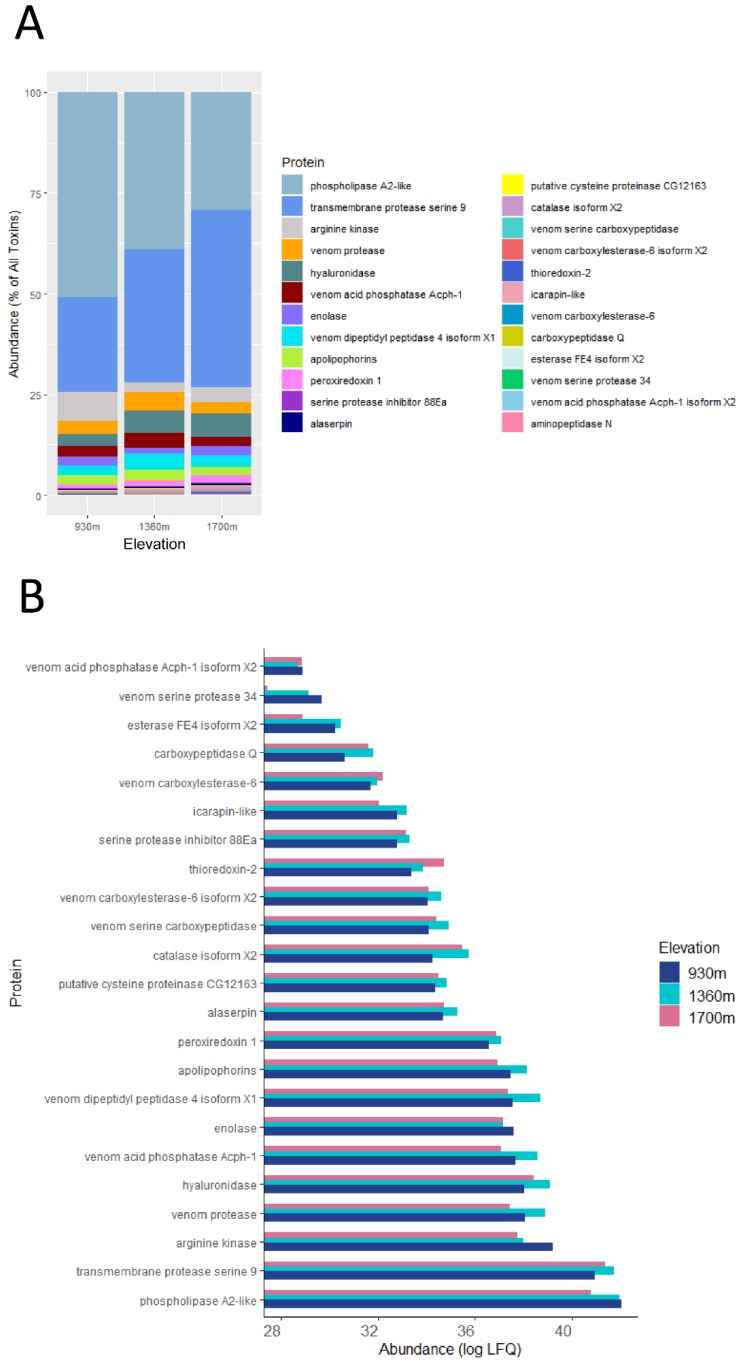
(**A**) Abundance of venom proteins for each site based on % of all toxins, (**B**) Bar plots showing abundances (LFQ) of venom proteins from the three sites represented in logarithmic scale (log2).

Upon proteomic analyses, a total of 1152 proteins were identified in at least one of the venom samples. Among the identified proteins, 24 venom proteins were selected for further analyses, based on their previous identification in bumblebee venom (Table S1). The molecular weights of these proteins ranged from 11 kilo Daltons (kDa) to 375 kDa. The total abundance of these toxins accounted for 49% of the venom proteome, with the rest being uncharacterized proteins, trace elements, and contaminants. The venom composition was visualized in a “parts to a whole chart”, where each toxin is presented as a percentage of total toxins (Figure 1A, Table S2). At all elevations, the toxin profile was predominantly composed of phospholipase A_2_-like (PLA_2_-like) and transmembrane protease serine 9 (TMPS9). We have conducted BLAST analyses on these two proteins to identify sequence similarities in the NCBI *Bombus* database. The protein sequence of PLA2-like was 98.89% identical with PLA_2_ (*B. hypocrita*) and 60% identical to PLA_2_ (*B. terrestris*). TMSP9 was structurally 91.19% identical with venom protease (*B. terrestris*) and 72.93% identical with venom serine protease Bi-VSP-like (*B. impatiens*). From low to high elevations, the venom toxin profile shifted from a PLA2-like-heavy profile to a TMPS9-heavy profile (Figure 1A). As illustrated by comparing the raw intensity values of each protein at each elevation (Figure 1B), the shift in toxin composition was mainly attributed to a gradual decrease in the level of PLA2-like towards high elevation. The total level of PLA2-like at 1700 m was 41.7% of the level observed at 930 m. Such a gradual decrease was also observed for arginine kinase and enolase. The levels of venom protease, venom acid phosphatase Acph-1, venom dipeptidyl peptidase 4 isoform X1, and apolipophorin were also higher at 930 m than at 1700 m. However, the decrease observed in these proteins was not gradual, with a peak observed at mid-elevation (1360 m). Thioredoxin-2 was the lone protein whose abundance gradually increased from 930 m to 1700 m. Although not gradual, the levels of TMPS9, hyaluronidase, serine protease inhibitor 88Ea, thioredoxin-2, venom serine carboxypeptidase, catalase isoform X2, putative cysteine proteinase CG12163, alaserpin, and peroxiredoxin all increased from 930 m to 1700 m. Altogether, these results suggest a shift in venom toxin composition along the elevation gradient.

### 2.2. Venom Toxins Show Differential Responses to Environmental Variations

**Figure 2:**
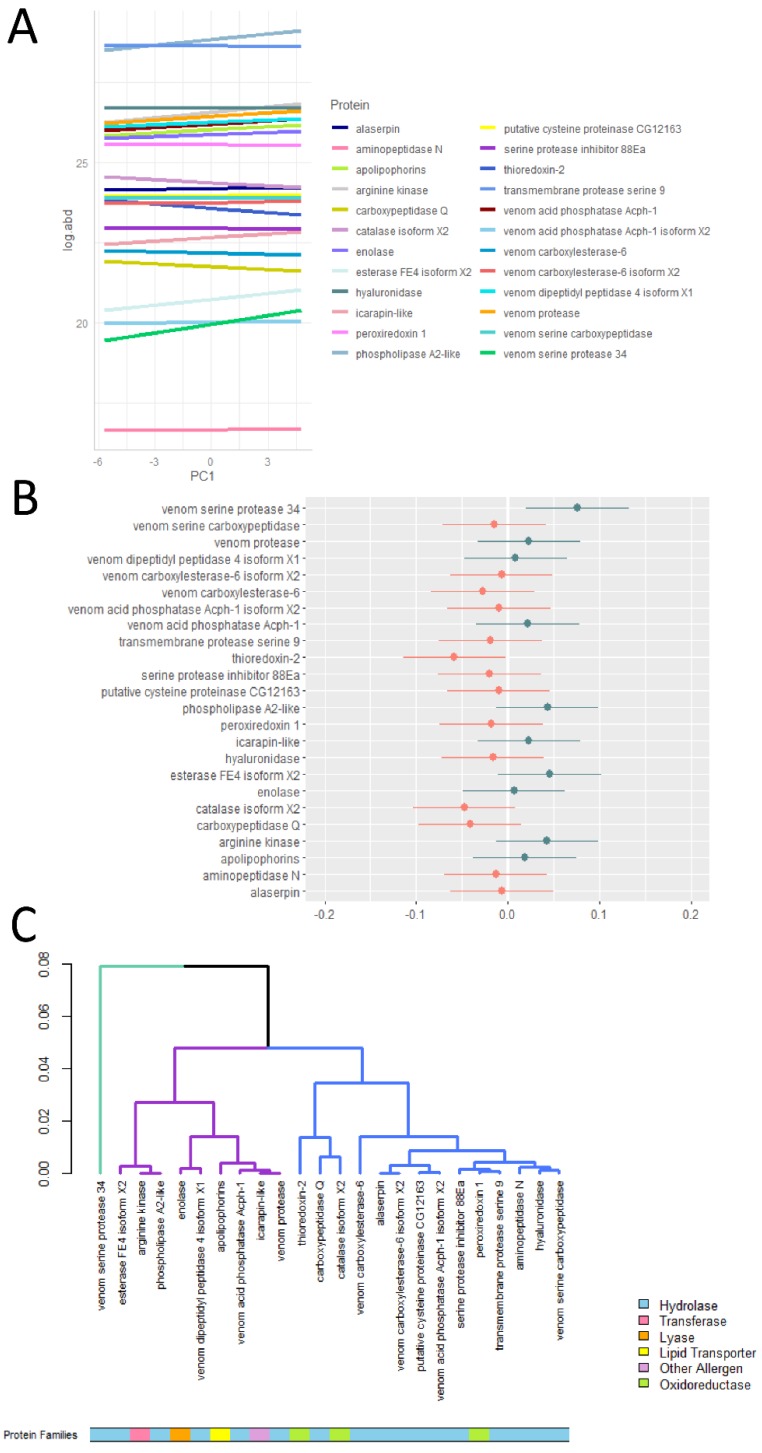
(**A**) Predicted changes of log-transformed protein abundances along the environmental gradient. (**B**) Departures from the main effect (a value of zero corresponds to the main effect, i.e., 0.014). Blue colors are indicative of proteins whose slope coefficients are higher that the main effect, whereas red colors are indicative of proteins whose slope coefficients are lower that the main effect. (**C)** Dendrogram performed on protein-wise slope coefficients showing the clustering of proteins with similar responses along the environmental gradient. Protein families of venom toxins are color-coded.

The first principal component axis performed on climatic variables (PC1; see methods) characterizes a temperature–humidity gradient, with negative values indicative of dry and warm habitats at low elevation and positive values indicative of wet and cold habitats at high elevation (Figure S1; Table S3). Using a mixed-effect model, we found a positive relationship between protein abundance and PC1 (estimated fixed-effect coefficient = 0.014). The relationship was however not significant (*p*-value = 0.72), presumably due to a lack of statistical power owing to the low sample size considered in this exploratory study. This relationship should thus be confirmed by the use of a larger dataset. The mixed-effect model further revealed departures from the main effect for several proteins (Figure 2A,B). For instance, PLA_2_-like, venom serine protease 34, esterase FE4 isoform X2, and arginine kinase—the major defensive toxins of bumblebee venom—displayed larger slope coefficients, suggesting that at low elevation (higher temperature), the abundance of these proteins increases more than what is observed on average. Although to a lesser extent, other defensive toxins such as venom protease, icarapin-like, venom dipeptidyl peptidase 4 isoform X1, apolipophorins, and enolase also presented larger slope coefficients than the main effect. On the other hand, thioredoxin-2, catalase isoform X2, and carboxypeptidase Q presented lower slope coefficients than the main effect, suggesting a decrease (or at least a lower increase) in abundance along the environmental gradient.

### 2.3. Defensive Venom Toxins Respond Similarly along the Environmental Gradient

The cluster analysis performed on protein-wise slope coefficients revealed three distinct clusters (Figure 2C). Venom serine protease 34, the protein showing the largest departure from the main effect, constituted the first cluster. The second cluster was composed of nine proteins that are pain-inducing and allergenic components of bumblebee venom. All of these proteins presented a slope coefficient higher than the main effect, suggesting a larger increase in abundance along the environmental gradient. The remaining third cluster was composed of proteins with allergenic or catalytic properties presenting lower slope coefficients than the main effect. Overall, this analysis suggests that the majority of defensive toxins show a particularly meaningful response toward an increase in abundance along the environmental gradient.

## 3. Discussion

Bumblebees are major pollinators of wild plants and critical players of ecosystems and biodiversity at high altitudes [31,47,48]. The documented venom plasticity and the thermoregulatory properties of bumblebees create a unique combination, making it possible to investigate the plastic response of venom toxins along an elevation gradient. In this exploratory study, our aim was to assess whether the venom composition of a bumblebee shows variation along an elevation gradient characterized by a gradient of temperature and humidity. Our results suggest that the venom toxin composition of *B. pascuorum* varies along the elevation gradient. One of the most prominent changes in the toxin composition was observed in one of the most potent venom toxins, PLA2-like, which gradually decreases towards high elevation.

In our proteomic analyses, the two most abundant proteins identified in the venom of *B. pascuorum* were PLA_2_-like and TMSP9. The protein sequence of PLA2-like is 98.89% identical to PLA_2_ (*B. hypocrita*) and 60% identical to PLA_2_ (*B. terrestris*), suggesting a functional similarity to PLA_2_. PLA_2_ is the major allergen found in bee venom and is responsible for hypersensitive reactions. It makes up to 10–12% of dry bee venom and induces mast cell maturation, anaphylactic shock, and neurotoxicity both in vitro and in vivo [49,50,51]. Interestingly, we have observed a major and a gradual decrease of PLA_2_-like levels toward high elevation. Meanwhile, the levels of thioredoxin-2, a member of an oxidoreductase family that negatively regulates PLA_2_ levels [52] gradually increased toward high elevation.

As the level of PLA_2_-like decreased with elevation, TMSP9 emerged as the most prominent venom toxin at high elevation. TMSP9 is structurally 91.19% identical with venom protease (*B. terrestris*) and 72.93% identical with venom serine protease Bi-VSP-like (*B. impatiens*), suggesting that it belongs to the bee venom protease family. These venom proteases are trypsins with a single serine protease domain which has allergenic properties and tryptic amidase activities [53]. Bi-VSPs, in particular, have been reported to exhibit fibrin(ogen)olytic activity during envenomation which inhibits blood coagulation, in turn facilitating the spread of other toxins in the bloodstream. Even though TMSP9 was the most abundant toxin at high elevation, its level only moderately increased from low to high elevation (1.34 fold higher at 1700 m vs. 930 m). Furthermore, we could neither observe a gradual increase in its abundance along the elevation gradient, nor detect a response along the environmental gradient. These results suggest that the major shift in *B. pascuorum* venom toxin profile toward high elevation is likely due to a decrease in PLA_2_-like.

Our proteomic analyses revealed that the levels of another member of the venom serine protease family, venom serine protease 34, gradually decreased toward high elevation. This was also the case for the allergens arginine kinase and enolase. In contrast, the levels of another allergen, venom carboxylesterase-6, gradually increased from low to high elevation, suggesting a heterogeneous variation in venom allergens. Toxicity assays are required to test whether the shift in toxin composition is associated with an altered potency of venom. However, as PLA_2_ is the major anaphylactic agent of bee venom [51], it can be speculated that the drastic decrease in its level at high elevation may render the venom less paralytic.

This is, to our knowledge, the first report on the characterization of bumblebee venom along an elevation gradient. The results of this study support the hypothesis that bumblebee venom shows plasticity along an elevation gradient. There could be several explanations to the observed plasticity. First, venom toxins could be directly influenced by environmental variables, as previously reported in predatory venoms, where protein levels have been shown to vary depending on temperature and seasonality [15,19,20,22,42]. A particular shift in an environmental factor may also alter the microhabitat of the bumblebee, and local adaption may affect venom composition [16]. Additionally, venom toxin levels may correlate with predator distributions and physiology due to the coevolutionary theory which suggests a phenotype matching occurring in predator–prey interactions. This is known as the escalating arms race and is well documented in predatory venoms [15,41,54,55,56]. As the frequency and the strength of biotic encounters decrease at high elevations due to colder climatic conditions [43,44,57,58,59,60], a lower predator pressure is expected to be exerted on bumblebees at high elevations. Therefore, the reduced levels of PLA_2_-like at high elevations may be a consequence of energy expenditure modulation. Such a phenomenon has been documented for plants which reduce their chemical defense at high elevation [59,60]. This may also hold true for certain components of the venom; whose production is highly energy-consuming for its host [5]. This, in turn, could bring a reduced defense at high elevation.

The influence of dietary differences on venom plasticity has mostly been documented for predatory venomous animals, as they use venom to capture prey [41]. The impact of diet on the venom composition in defensive venomous animals is elusive. In this study, we tried to minimize the potential influence of diet by collecting bumblebees while they were foraging on *Trifolium* spp. However, we cannot exclude the possibility of bumblebees reaching other plant species. In future studies, the direct impact of diet on bumblebee venom may be determined through controlled experiments on caged or laboratory-reared bumblebees. This study was a first exploratory step toward a better understanding of the processes (both biotic and abiotic) affecting bumblebee venom composition along an elevation gradient. Technically, considering the complex nature of venom, additional methods such as venom gland transcriptomics should be used to complement the identified proteins. In future studies, the influence of population genetics on venom composition should also be considered. This study may also be complemented by including other bumblebee species and sampling a larger number of individuals and sites. This would enable the use of spatial modelling approaches to predict current and future distributions of venom proteins, for example, using different global climate change scenarios. If a general pattern is observed, this, in turn, could be used as an indicator of bumblebee vulnerability.

## 4. Conclusions

In the present study, we report the first findings on the influence of environmental factors on bumblebee venom variation, and particularly the alteration of venom and thus reduced defense towards higher elevations. These results provide evidence for the dynamic nature of major defensive toxins and suggest that the toxins may be vulnerable to drastic environmental changes.

## 5. Materials and Methods

### 5.1. Study Area and Bumblebee Sampling

**Figure 3:**
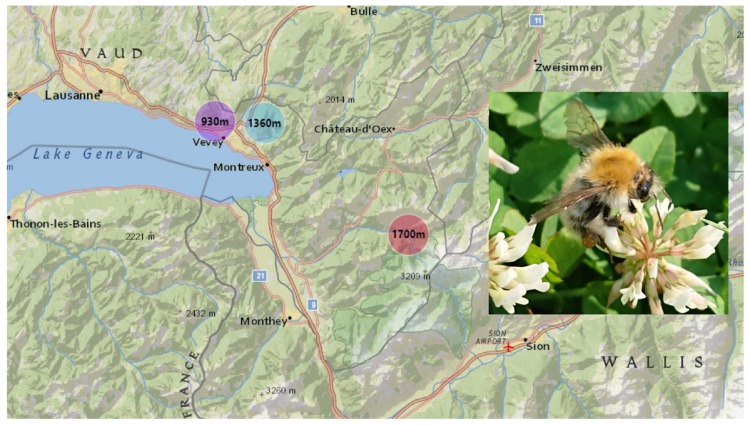
Sampling area in the western Swiss Alps: 930 m: Mont-Pèlerin, 1360 m: Les Pleiades, 1700 m: Col du Pillon, Les Diablerets. *B. pascuorum* photo taken by N. P. Barkan.

The study area is located in the Western Swiss Alps, a priority area for interdisciplinary research (see [61]). To assess compositional differences in venom composition along an elevation gradient, we sampled six bumblebees at three sampling sites, located at different altitudes (930 m, 1360 m, and 1700 m; Figure 3). Sampling took place in early September during active hours (12:00–17:00). We focused our study on one species (*B. pascuorum*), a widespread species inhabiting different habitats and a wide range of altitudes. Populations of *B. pascuorum* with similar coat colors were collected using nets. Bumblebee specimens were kept alive in ventilated tubes and brought to the laboratory, where they were sedated at −20 °C for 15 min.

### 5.2. Venom Extraction and Protein Identification of Venom Components

Venom was collected from a total of 18 bumblebee samples (six from each elevation) by removing the venom reservoir and collecting the venom immediately in protein Eppendorf LoBind^®^ tubes (Eppendorf AG, Hamburg, Germany) and stored at −80 °C until use. Venom samples were analyzed using shot-gun proteomic approaches. Venom samples from each elevation were pooled. A total of 8 µL of each sample was loaded on a sodium dodecyl sulfate–polyacrylamide gel (SDS-PAGE, 15%), and the appearing six bands were cut after gel migration (Figure S2). Bands were digested separately, and the corresponding proteins were analyzed on a Orbitrap Fusion tribrid Mass Spectrometer (Thermo Fisher, Bremen, Germany) (parameters: 65 min gradient, MS/MS IT HCD). The remaining venom sample (~2 µL) was digested in solution using miST protocol, which included manual trypsin digestion with reduction and carboxyamidation steps. The obtained elute was directly injected on Fusion MS without fractionation (parameters: 140 min gradient, MS/MS IT HCD (low resolution) or MS/MS OT HCD or EThcD (high resolution). Label-free quantification (LFQ intensity) values were obtained for each identified venom toxin. We used these values as a proxy for protein abundances. For database research, as a *B. pascuorum* database does not exist, MS/MS spectra obtained from *B. pascuorum* venom were submitted to MASCOT search engine using the closest homologues, *B. impatiens* (v 2.1) and *B. terrestris* (v 1.0) databases (custom-made databases containing usual contaminants such as digestion enzymes and human keratins). As the *B. terrestris* (v 1.0) database yielded more toxin components, it was used in further analyses. Scaffold (version 4.8.7, Proteome Software Inc, Portland, OR, USA) was used to validate peptide and protein identifications and visualize data. PEAKS software (Bioinformatics Solutions Inc, Waterloo, ON, Canada) was used on high-resolution data (from in solution digestion). Protein vs. protein alignment was conducted on NCBI protein BLAST suite (https://blast.ncbi.nlm.nih.gov/Blast.cgi) [62] using Accession Number (Table S1) as query and *Bombus* (taxid: 28641) as database.

### 5.3. Environmental Data

Nineteen environmental variables were extracted from the CHclim25 database, containing highly resolved (25 m) environmental information [63]. From this set of variables (Table S3), we conducted a principal component analysis (PCA [64]; ade4 package [65]) and extracted the first axis which explained 98.9% of the variance for further analyses. A two-dimensional correlation circle depicting the contribution of the variables to the two PCA components is shown in Figure S1A. The correlation between variables varied from −1 to +1 (Figure S1B).

### 5.4. Statistical Analyses

From the proteomic analysis, we obtained a total of 72 observations corresponding to the LFQ intensity (abundance) values of 24 proteins obtained at the three elevations. To assess differential responses of venom proteins across the environmental gradient, we used mixed-effect models (using the lmer function in the lme4 package [66] in R [67]) with the log-transformed LFQ intensity values as the dependent variable and the first environmental PCA axis (PC1) as the independent variable. Protein ID was included in the model as a random effect on both the intercept and the slope coefficient. From this analysis, we extracted protein-wise slope coefficients (i.e., the random effects) to assess departures from the main effect (representing overall changes in protein abundance), to determine the extent to which the abundances of proteins changed along the environmental gradient. Finally, we conducted a cluster analysis using the average method [68] across all protein-wise slope coefficients to assess whether proteins can be clustered on the basis of their response along the environmental gradient. 

## Supplementary Materials

The following are available online at https://www.mdpi.com/2072-6651/12/1/4/s1, Figure S1: A. Correlation circle representing the contribution of environmental variables on PC1 and PC2 (dim 1: PC1, dim 2: PC2); B. Correlation matrix plot among environmental variables (bar represents Pearson’s correlation coefficients). Full names of the environmental variables are shown in Table S3, Figure S2: SDS-PAGE gel images of venom samples from 930 m, 1360 m, and 1700 m. A, B, C, D, E, and F are protein bands that were further analyzed. Table S1: List of 24 venom proteins identified from *B. pascuorum* which are selected for further analyses. Table S2: LFQ intensity values of the 24 venom proteins identified from *B. pascuorum*. Table S3. List of environmental variables extracted from the CHclim25 database.

## References

[1] Calvete J.J. (2009). Venomics: Digging into the evolution of venomous systems and learning to twist nature to fight pathology. J. Proteom..

[2] Schmidt J.O. (1982). Biochemistry of insect venoms. Annu. Rev. Entomol..

[3] Whittington C.M., Belov K. (2014). Tracing monotreme venom evolution in the genomics era. Toxins.

[4] Casewell N.R., Wüster W., Vonk F.J., Harrison R.A., Fry B.G. (2013). Complex cocktails: The evolutionary novelty of venoms. Trends Ecol. Evol..

[5] Arbuckle K., Malhotra A. (2017). Evolutionary Context of Venom in Animals. Evolution of Venomous Animals and Their Toxins.

[6] Fry B.G., Scheib H., van der Weerd L., Young B., McNaughtan J., Ramjan S.F., Vidal N., Poelmann R.E., Norman J.A. (2008). Evolution of an arsenal: Structural and functional diversification of the venom system in the advanced snakes (Caenophidia). MCP.

[7] Fry B.G., Vidal N., Norman J.A., Vonk F.J., Scheib H., Ramjan S.F., Kuruppu S., Fung K., Hedges S.B., Richardson M.K. (2006). Early evolution of the venom system in lizards and snakes. Nature.

[8] Fry B.G., Vidal N., van der Weerd L., Kochva E., Renjifo C. (2009). Evolution and diversification of the Toxicofera reptile venom system. J. Proteom..

[9] Zhang Y. (2015). Why do we study animal toxins?. Zool. Res..

[10] Gibbs H.L., Rossiter W. (2008). Rapid Evolution by Positive Selection and Gene Gain and Loss: PLA2 Venom Genes in Closely Related Sistrurus Rattlesnakes with Divergent Diets. J. Mol. Evol..

[11] Westermann F.L., McPherson I.S., Jones T.H., Milicich L., Lester P.J. (2015). Toxicity and utilization of chemical weapons: Does toxicity and venom utilization contribute to the formation of species communities?. Ecol. Evol..

[12] Rodríguez de la Vega R.C., Schwartz E.F., Possani L.D. (2010). Mining on scorpion venom biodiversity. Toxicon Off. J. Int. Soc. Toxinol..

[13] Columbus-Shenkar Y.Y., Sachkova M.Y., Macrander J., Fridrich A., Modepalli V., Reitzel A.M., Sunagar K., Moran Y. (2018). Dynamics of venom composition across a complex life cycle. eLife.

[14] Gangur A.N., Smout M., Liddell M.J., Seymour J.E., Wilson D., Northfield T.D. (2017). Changes in predator exposure, but not in diet, induce phenotypic plasticity in scorpion venom. Proc. Biol. Sci..

[15] Holding M.L., Biardi J.E., Gibbs H.L. (2016). Coevolution of venom function and venom resistance in a rattlesnake predator and its squirrel prey. Proc. Biol. Sci..

[16] Daltry J.C., Wüster W., Thorpe R.S. (1996). Diet and snake venom evolution. Nature.

[17] Gibbs H.L., Mackessy S.P. (2009). Functional basis of a molecular adaptation: Prey-specific toxic effects of venom from Sistrurus rattlesnakes. Toxicon Off. J. Int. Soc. Toxinol..

[18] Gibbs H.L., Sanz L., Chiucchi J.E., Farrell T.M., Calvete J.J. (2011). Proteomic analysis of ontogenetic and diet-related changes in venom composition of juvenile and adult Dusky Pigmy rattlesnakes (*Sistrurus miliarius* barbouri). J. Proteom..

[19] O’Hara E.P., Caldwell G.S., Bythell J. (2018). Equistatin and equinatoxin gene expression is influenced by environmental temperature in the sea anemone Actinia equina. Toxicon Off. J. Int. Soc. Toxinol..

[20] Strickland J.L., Smith C.F., Mason A.J., Schield D.R., Borja M., Castaneda-Gaytan G., Spencer C.L., Smith L.L., Trapaga A., Bouzid N.M. (2018). Evidence for divergent patterns of local selection driving venom variation in Mojave Rattlesnakes (Crotalus scutulatus). Sci. Rep..

[21] Winter K.L., Isbister G.K., McGowan S., Konstantakopoulos N., Seymour J.E., Hodgson W.C. (2010). A pharmacological and biochemical examination of the geographical variation of *Chironex fleckeri* venom. Toxicol. Lett..

[22] Zancolli G., Calvete J.J., Cardwell M.D., Greene H.W., Hayes W.K., Hegarty M.J., Herrmann H.-W., Holycross A.T., Lannutti D.I., Mulley J.F. (2019). When one phenotype is not enough: Divergent evolutionary trajectories govern venom variation in a widespread rattlesnake species. Proc. R. Soc. B.

[23] Huber J.T. (2009). Biodiversity of Hymenoptera. Insect Biodiversity.

[24] Klopfstein S., Vilhelmsen L., Heraty J.M., Sharkey M., Ronquist F. (2013). The Hymenopteran Tree of Life: Evidence from Protein-Coding Genes and Objectively Aligned Ribosomal Data. PLoS ONE.

[25] Cameron S.A., Williams P.H. (2003). Phylogeny of bumble bees in the New World subgenus Fervidobombus (Hymenoptera: Apidae): Congruence of molecular and morphological data. Mol. Phylogenetics Evol..

[26] Heinrich B. (1979). Resource heterogeneity and patterns of movement in foraging bumblebees. Oecologia.

[27] Pradervand J.N., Pellissier L., Rossier L., Dubuis A., Guisan A., Cherix D. (2011). Diversity of bumblebees (Bombus Latreille, Apidae) in the Alps of the canton Vaud (Switzerland). Bull. Société Entomol. Suisse.

[28] Woodard S.H. (2017). Bumble bee ecophysiology: Integrating the changing environment and the organism. Curr. Opin. Insect Sci..

[29] Williams P.H., Cameron S.A., Hines H.M., Cederberg B., Rasmont P. (2008). A simplified subgeneric classification of the bumblebees (genus Bombus). Apidologie.

[30] Biella P., Bogliani G., Cornalba M., Manino A., Neumayer J., Porporato M., Rasmont P., Milanesi P. (2017). Distribution patterns of the cold adapted bumblebee Bombus alpinus in the Alps and hints of an uphill shift (Insecta: Hymenoptera: Apidae). J. Insect Conserv..

[31] Free J. (1993). Insect Pollination of Crops.

[32] Dirnböck T., Essl F., Rabitsch W. (2011). Disproportional risk for habitat loss of high-altitude endemic species under climate change. Glob. Chang. Biol..

[33] Nogués-Bravo D., Araújo M.B., Errea M.P., Martínez-Rica J.P. (2007). Exposure of global mountain systems to climate warming during the 21st Century. Glob. Environ. Chang..

[34] Von Reumont B.M., Campbell L.I., Jenner R.A. (2014). Quo vadis venomics? A roadmap to neglected venomous invertebrates. Toxins.

[35] Dutertre S., Jin A.H., Vetter I., Hamilton B., Sunagar K., Lavergne V., Dutertre V., Fry B.G., Antunes A., Venter D.J. (2014). Evolution of separate predation- and defence-evoked venoms in carnivorous cone snails. Nat. Commun..

[36] Barkan N.P., Bayazit M.B., Ozel Demiralp D. (2017). Proteomic Characterization of the Venom of Five Bombus (Thoracobombus) Species. Toxins.

[37] Van Vaerenbergh M., Debyser G., Smagghe G., Devreese B., de Graaf D.C. (2015). Unraveling the venom proteome of the bumblebee (Bombus terrestris) by integrating a combinatorial peptide ligand library approach with FT-ICR MS. Toxicon Off. J. Int. Soc. Toxinol..

[38] Argiolas A., Pisano J.J. (1985). Bombolitins, a new class of mast cell degranulating peptides from the venom of the bumblebee *Megabombus pennsylvanicus*. J. Biol. Chem..

[39] Schmidt J.O. (2019). The Insect Sting Pain Scale: How the Pain and Lethality of Ant, Wasp, and Bee Venoms Can Guide the Way for Human Benefit. Preprints.

[40] Goulson D., O’Connor S., Park K.J. (2018). The impacts of predators and parasites on wild bumblebee colonies. Ecol. Entomol..

[41] Holding M.L., Margres M.J., Rokyta D.R., Gibbs H.L. (2018). Local prey community composition and genetic distance predict venom divergence among populations of the northern Pacific rattlesnake (*Crotalus oreganus*). J. Evol. Biol..

[42] Junior R.S.F., Sciani J.M., Marques-Porto R., Junior A.L., Orsi R.d.O., Barraviera B., Pimenta D.C. (2010). Africanized honey bee (*Apis mellifera*) venom profiling: Seasonal variation of melittin and phospholipase A 2 levels. Toxicon Off. J. Int. Soc. Toxinol..

[43] Romero G.Q., Gonçalves-Souza T., Kratina P., Marino N.A.C., Petry W.K., Sobral-Souza T., Roslin T. (2018). Global predation pressure redistribution under future climate change. Nat. Clim. Chang..

[44] Pennings S.C., Silliman B.R. (2005). Linking Biogeography and Community Ecology: Latitudinal Variation in Plant-Herbivore Interaction Strength. Ecology.

[45] Roslin T., Hardwick B., Novotny V., Petry W.K., Andrew N.R., Asmus A., Barrio I.C., Basset Y., Boesing A.L., Bonebrake T.C. (2017). Higher predation risk for insect prey at low latitudes and elevations. Science.

[46] Morgenstern D., King G.F. (2013). The venom optimization hypothesis revisited. Toxicon Off. J. Int. Soc. Toxinol..

[47] Goulson D., Lye G.C., Darvill B. (2008). Decline and conservation of bumble bees. Annu. Rev. Entomol..

[48] Williams P., Colla S., Xie Z. (2009). Bumblebee Vulnerability: Common Correlates of Winners and Losers across Three Continents. Conserv. Biol..

[49] Clapp L.E., Klette K.L., DeCoster M.A., Bernton E., Petras J.M., Dave J.R., Laskosky M.S., Smallridge R.C., Tortella F.C. (1995). Phospholipase A2-induced neurotoxicity in vitro and in vivo in rats. Brain Res..

[50] Lee G., Bae H. (2016). Bee Venom Phospholipase A2: Yesterday’s Enemy Becomes Today’s Friend. Toxins.

[51] Perez-Riverol A., Lasa A.M., Dos Santos-Pinto J.R.A., Palma M.S. (2019). Insect venom phospholipases A1 and A2: Roles in the envenoming process and allergy. Insect Biochem. Mol. Biol..

[52] Lozano R.M., Yee B.C., Buchanan B.B. (1994). Thioredoxin-linked reductive inactivation of venom neurotoxins. Arch. Biochem. Biophys..

[53] Winningham K.M., Fitch C.D., Schmidt M., Hoffman D.R. (2004). Hymenoptera venom protease allergens. J. Allergy Clin. Immunol..

[54] Brodie E.D., Ridenhour B.J., Brodie E.D. (2002). The evolutionary response of predators to dangerous prey: Hotspots and coldspots in the geographic mosaic of coevolution between garter snakes and newts. Evol. Int. J. Org. Evol..

[55] Hanifin C.T., Brodie E.D., Brodie E.D. (2008). Phenotypic mismatches reveal escape from arms-race coevolution. PLoS Biol..

[56] Pekar S., Liznarova E., Bocanek O., Zdrahal Z. (2018). Venom of prey-specialized spiders is more toxic to their preferred prey: A result of prey-specific toxins. J. Anim. Ecol..

[57] Dobzhansky T. (1950). Evolution in the tropics. Am. Sci..

[58] MacArthur R.H. (1972). Geographical Ecology: Patterns in the Distribution of Species.

[59] Pellissier L., Fiedler K., Ndribe C., Dubuis A., Pradervand J.-N., Guisan A., Rasmann S. (2012). Shifts in species richness, herbivore specialization, and plant resistance along elevation gradients. Ecol. Evol..

[60] Descombes P., Marchon J., Pradervand J.-N., Bilat J., Guisan A., Rasmann S., Pellissier L. (2017). Community-level plant palatability increases with elevation as insect herbivore abundance declines. J. Ecol..

[61] RECHALP: A Geodatabase of Scientific Metadata for the Vaud Alps. http://rechalp.unil.ch.

[62] NCBI Protein Blast. https://blast.ncbi.nlm.nih.gov/Blast.cgi.

[63] Broennimann O. (2018). CHclim25: A High Spatial and Temporal Resolution Climate Dataset for Switzerland.

[64] Jolliffe I., Lovric M. (2011). Principal Component Analysis. International Encyclopedia of Statistical Science.

[65] Dray D. (2007). The ade4 Package: Implementing the Duality Diagram for Ecologists. J. Stat. Softw..

[66] Bates D., Mächler M., Bolker B., Walker S. (2015). Fitting Linear Mixed-Effects Models Using lme4. J. Stat. Softw..

[67] R Foundation for Statistical Computing (2018). R: A Language and Environment for Statistical Computing.

[68] Jain A.K., Dubes R.C. (1988). Algorithms for Clustering Data.

