# Supplementary material for: Alteration of Bumblebee Venom Composition toward Higher Elevation
Source: Toxins (Basel). 2019 Dec 19;12(1):4. doi: 10.3390/toxins12010004 (PMC7020474; doi:10.3390/toxins12010004)
Supplement: Supplementary file 1 [file toxins-12-00004-s001.pdf]

Supplementary Materials: Alteration of Bumblebee Venom Composition toward Higher Elevation

Nezahat Pınar Barkan, Mathieu Chevalier, Jean-Nicolas Pradervand and Antoine Guisan

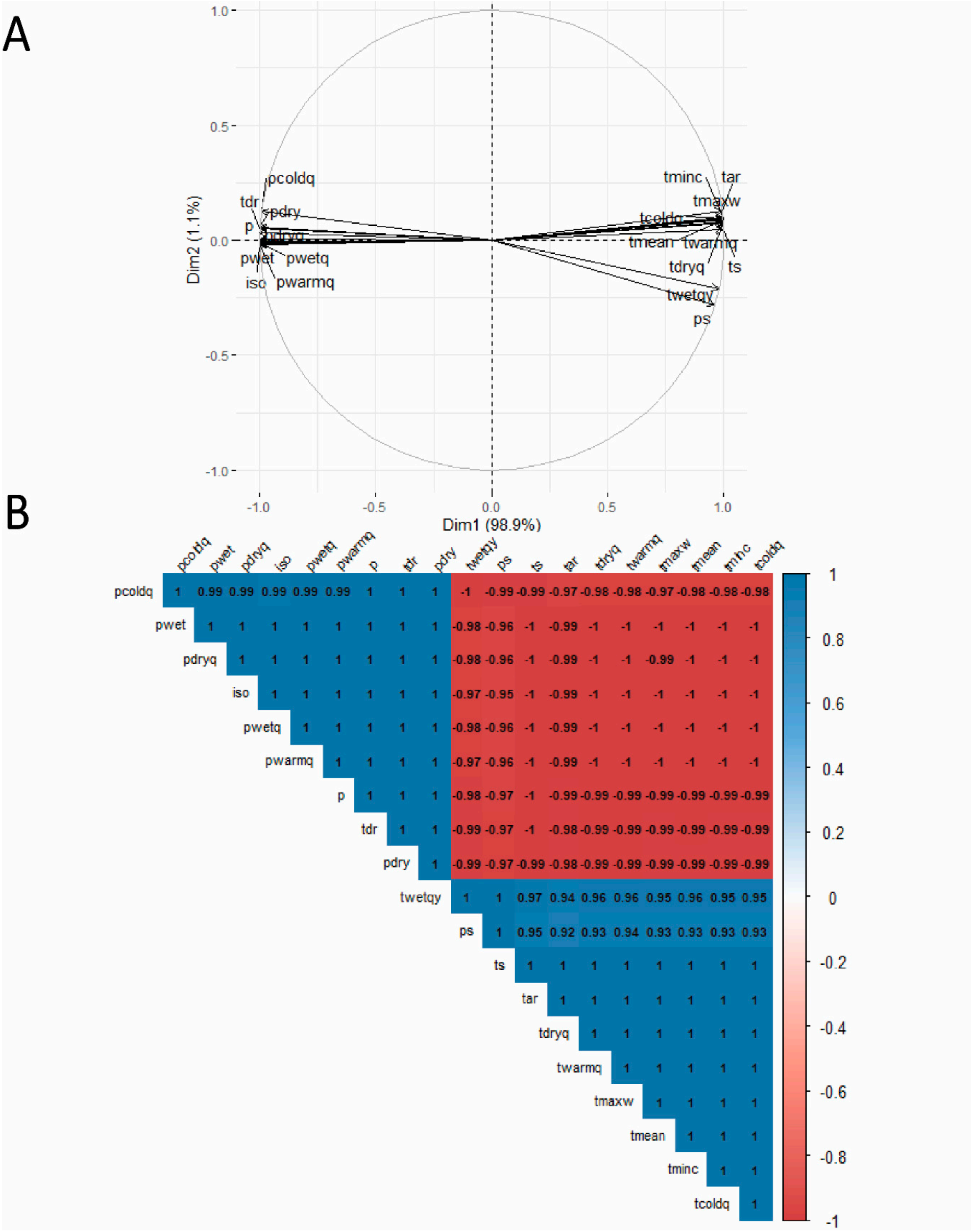

Figure S1. (**A**) Correlation circle representing the contribution of environmental variables on PC1 and PC2 (dim 1: PC1, dim 2: PC2) (**B**) Correlation matrix plot among environmental variables (bar represents Pearson's correlation coefficients). Full names of the environmental variables are shown in Table S3.

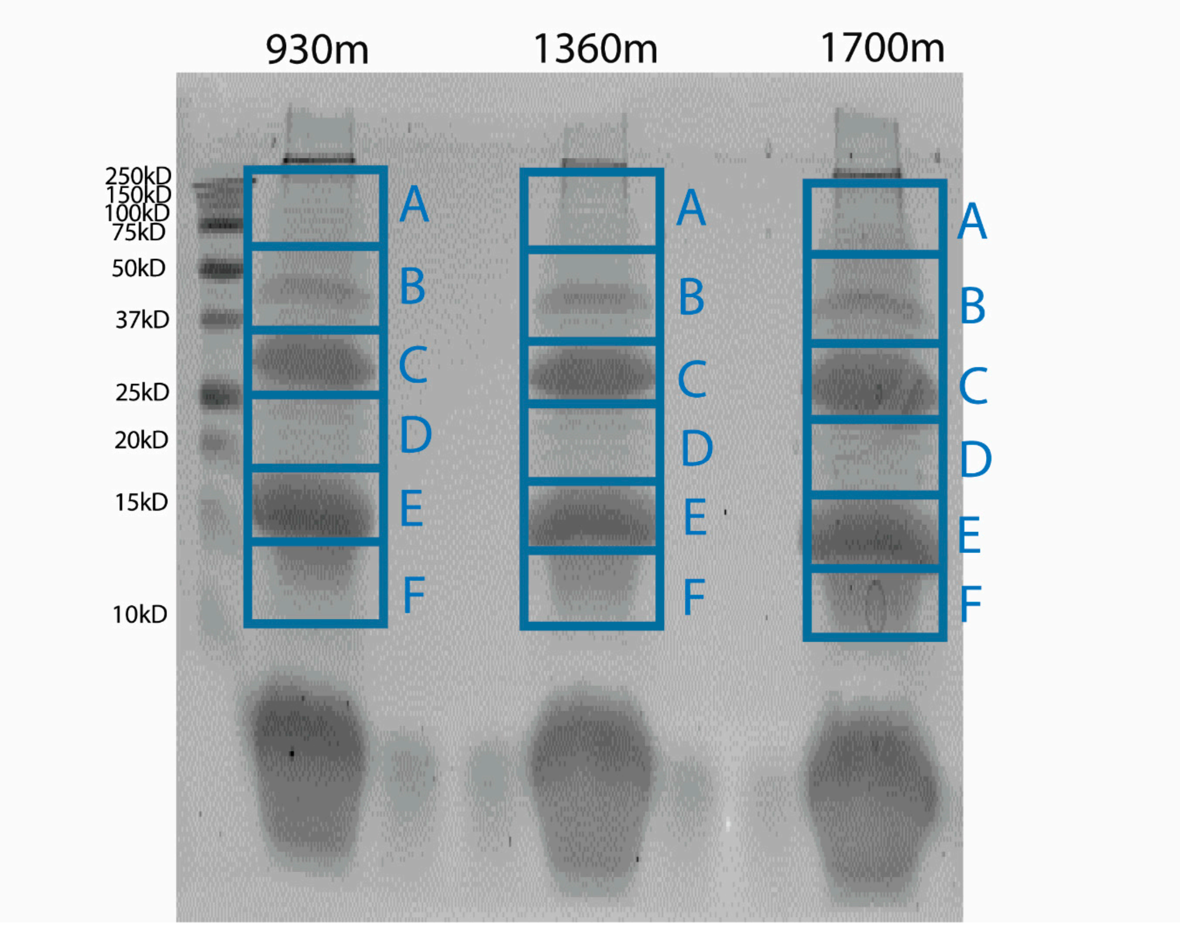

Figure S2. SDS-PAGE gel images of venom samples from 930 m, 1360 m and 1700 m. A, B, C, D, E and F are protein bands that were further analyzed.

Table S1. List of 24 venom proteins identified from *B. pascuorum* which are selected for further analyses.

Protein	Accession Number	Molecular Weight (kDa)	Score	Peptides	Razor + Unique Peptides	Unique peptides	Intensity	MS/MS Count	MS/MS Count 930 m	MS/MS Count 1360m	MS/MS Count 1700 m
Phospholipase A2-like	XP_012170987.1	21	318.2	15	15	15	8.0×10^{11}	618	240	159	219
Transmembrane protease serine 9	XP_020718442.1	67	323.3	21	21	13	1.23×10^{12}	856	315	268	273
Arginine kinase	XP_003401502.1	42	323.3	27	27	27	4.93×10^{11}	631	250	178	203
Venom protease	XP_012163516.1	33	151.5	8	8	8	1.34×10^{11}	91	26	30	35
Hyaluronidase	XP_012171522.1	44	323.3	28	28	28	6.26×10^{11}	488	142	175	171
Venom acid phosphatase Acph-1	XP_012176424.1	48	323.3	28	28	28	4.16×10^{11}	317	103	115	99
Enolase	XP_012170810.1	47	323.3	24	24	24	2.60×10^{11}	427	150	131	146
Venom dipeptidyl peptidase 4 isoform X1	XP_003399213.1;XP_020721340.1	89	323.3	33	33	33	5.95×10^{11}	627	209	229	189
Apolipophorins	XP_003397320.1	375	323.3	109	109	109	4.48×10^{11}	693	237	256	200
Peroxiredoxin 1	XP_012172464.1;XP_012172463.1	22	323.3	14	14	13	9.05×10^{10}	175	46	53	76
Venom protease-like	XP_020723946.1	12	18.55	5	1	1	2.51×10^{10}	34	10	13	11
Alaserpin	XP_012169463.2	45	221.9	11	11	6	4.01×10^{10}	100	30	35	35
Putative cysteine proteinase CG12163	XP_012163961.1	100	323.3	21	21	21	3.05×10^{10}	123	43	44	36
Catalase isoform X2	XP_012164575.1;XP_012164574.1	67	323.3	18	18	18	7.31×10^{10}	209	65	73	71
Venom serine carboxypeptidase	XP_003400098.1	54	323.3	16	16	16	5.25×10^{10}	168	60	57	51
Venom carboxylesterase-6	XP_012176619.2;XP_020724240.1	60	323.3	16	16	15	4.83×10^{10}	123	36	43	44
Venom carboxylesterase-6 isoform X2	XP_012176619.2;XP_020724240.1	60	323.3	16	16	15	4.83×10^{10}	123	36	43	44
Thioredoxin-2	XP_012164907.1	11	70.78	6	6	6	7.83×10^{10}	36	15	13	8
Icarapin-like	XP_003396228.1	25	76.62	6	6	6	6.79×10^{10}	74	31	22	21
Carboxypeptidase Q	XP_020722482.1;XP_020722481.1 ;XP_003401007.1	53	110.9	9	9	9	7.32×10^{10}	40	11	14	15
Esterase FE4 isoform X2	XP_020719342.1;XP_003396911.1	57	73.34	8	8	8	2.25×10^{10}	27	11	9	7
Venom serine protease 34	XP_003402441.1	45	53.31	4	4	4	5.04×10^{10}	6	2	3	1
Venom acid phosphatase Acph-1 isoform X2	XP_003399644.1;XP_012170162.1	44	64.65	5	5	5	8.83×10^{10}	12	4	2	6
Aminopeptidase N	XP_003402607.1	112	14.59	2	2	2	5.04×10^{10}	2	0	1	1
Serine protease inhibitor 88Ea	XP_003398424.1	50	323.3	19	19	19	1.69×10^{10}	89	26	32	31

Table S2. LFQ intensity values of the 24 venom proteins identified from *B. pascuorum*.

Protein	LFQ Intensity_930 m	LFQ Intensity_1360 m	LFQ Intensity_1700 m
phospholipase A2-like	4.50293×10^{12}	4.266×10^{12}	1.876×10^{12}
transmembrane protease serine 9	2.08763×10^{12}	3.575×10^{12}	2.803×10^{12}
arginine kinase	6.40319×10^{11}	2.762×10^{11}	2.329×10^{11}
venom protease	2.86352×10^{11}	5.073×10^{11}	1.841×10^{11}
hyaluronidase	2.80459×10^{11}	5.904×10^{11}	3.66×10^{11}
venom acid phosphatase Acph-1	2.20502×10^{11}	4.132×10^{11}	1.46×10^{11}
enolase	2.08607×10^{11}	1.532×10^{11}	1.548×10^{11}
venom dipeptidyl peptidase 4 isoform X1	2.04456×10^{11}	4.389×10^{11}	1.738×10^{11}
apolipophorins	1.92491×10^{11}	3.0394×10^{11}	1.3111×10^{11}
peroxiredoxin 1	1.01663×10^{11}	1.4427×10^{11}	1.2815×10^{11}
alaserpin	2.73980×10^{10}	4.1836×10^{10}	2.839×10^{10}
putative cysteine proteinase CG12163	2.21840×10^{10}	3.1003×10^{10}	2.4076×10^{10}
catalase isoform X2	2.02472×10^{10}	5.7906×10^{10}	4.7362×10^{10}
venom serine carboxypeptidase	1.85796×10^{10}	3.2126×10^{10}	2.2602×10^{10}
venom carboxylesterase-6 isoform X2	1.78970×10^{10}	2.6312×10^{10}	1.8464×10^{10}
thioredoxin-2	1.13660×10^{10}	1.5537×10^{10}	2.8338×10^{10}
serine protease inhibitor	7.40810×10^{9}	1.079×10^{10}	9.583×10^{9}
icarapin-like	7.42632×10^{9}	9.765×10^{9}	4.446×10^{9}
venom carboxylesterase-6	3.52995×10^{9}	4.18×10^{9}	4.992×10^{9}
carboxypeptidase Q	1.66340×10^{9}	3.83×10^{9}	3.315×10^{9}
esterase FE4 isoform X2	1.27690×10^{9}	1.512×10^{9}	5.0231×10^{8}
venom serine protease 34	8.58335×10^{8}	5.9901×10^{8}	1.8309×10^{8}
venom acid phosphatase Acph-1 isoform X2	5.07559×10^{8}	4.4462×10^{8}	4.9539×10^{8}
Aminopeptidase N	1.16191×10^{7}	2.9373×10^{7}	1.3943×10^{7}

Table S3. List of environmental variables extracted from the CHclim25 database.

Climatic Variable	Definition (unit)	Elevation	Value
tmean (BIO1)	Annual mean temperature (°C)	930 m	8.83
		1360 m	6.69
		1700 m	4.07
tdr (BIO2)	Mean diurnal range (°C)	930 m	7.74
		1360 m	7.92
		1700 m	8.29
iso (BIO3)	Isothermality (%)	930 m	30.71
		1360 m	32.26
		1700 m	34.68
ts (BIO4)	Temperature seasonality (%)	930 m	650.81
		1360 m	621.16
		1700 m	579.02
tmaxw (BIO5)	Maximum temperature of warmest month (°C)	930 m	22.82
		1360 m	20.14
		1700 m	17.01
tminc (BIO6)	Minimum temperature of coldest month (°C)	930 m	−2.37
		1360 m	−4.42
		1700 m	−6.88
tar (BIO7)	Temperature annual range (°C)	930 m	25.19
		1360 m	24.56

		1700 m	23.89
		930 m	15.13
twetqy (BIO8)	Mean temperature of wettest quarter (°C)	1360 m	14.45
		1700 m	11.26
		930 m	4.34
tdryq (BIO9)	Mean temperature of driest quarter (°C)	1360 m	2.11
		1700 m	−0.69
twarmq (BIO10)		930 m	16.95
	Mean temperature of warmest quarter (°C)	1360 m	14.45
		1700 m	11.26
		930 m	2.29
tcoldq (BIO11)	Mean temperature of coldest quarter (°C)	1360 m	0.36
		1700 m	−1.96
		930 m	1445.72
p (BIO12)	Annual precipitation (mm)	1360 m	1606.5
		1700 m	1916
		930 m	145.11
pwet (BIO13)	Precipitation of wettest month (mm)	1360 m	161.94
		1700 m	190.01
		930 m	89.32
pdry (BIO14)	Precipitation of driest month (mm)	1360 m	101.7
		1700 m	127.95
		930 m	14.37
ps (BIO15)	Precipitation of seasonality (%)	1360 m	14.18
		1700 m	12.53
		930 m	410.71
pwetq (BIO16)	Precipitation of wettest quarter (mm)	1360 m	466.68
		1700 m	557.93
		930 m	305.94
pdryq (BIO17)	Precipitation of driest quarter (mm)	1360 m	348.53
		1700 m	421.13
		930 m	409.53
pwarmq (BIO18)	Precipitation of warmest quarter (mm)	1360 m	466.68
		1700 m	557.93
		930 m	308.36
pcoldq (BIO19)	Precipitation of coldest quarter (mm)	1360 m	349.59
		1700 m	467.3
